# Three-Dimensional Fractal Geometry for Gas Permeation in Microchannels

**DOI:** 10.3390/mi9020045

**Published:** 2018-01-27

**Authors:** Magdalena Malankowska, Stefan Schlautmann, Erwin J. W. Berenschot, Roald M. Tiggelaar, Maria Pilar Pina, Reyes Mallada, Niels R. Tas, Han Gardeniers

**Affiliations:** 1 Department of Chemical & Enviromental Engineering, Nanoscience Institute of Aragon, University of Zaragoza, Edif I+D+i, Campus Río Ebro, C/Mariano Esquillor, s/n, 50018 Zaragoza, Spain; magdalena.malankowska@gmail.com (M.M.); mapina@unizar.es (M.P.P.); 2 Mesoscale Chemical Systems, MESA+ Institute for Nanotechnology, University of Twente, P.O. Box 217, 7500 AE Enschede, The Netherlands; s.schlautmann@utwente.nl (S.S.); j.w.berenschot@utwente.nl (E.J.W.B.); r.m.tiggelaar@utwente.nl (R.M.T.); n.r.tas@utwente.nl (N.R.T.); j.g.e.gardeniers@utwente.nl (H.G.); 3 NanoLab cleanroom, MESA+ Institute for Nanotechnology, University of Twente, P.O. Box 217, 7500 AE Enschede, The Netherlands

**Keywords:** nanonozzles, gas permeation, fractal geometry, corner lithography, integrated membrane chip

## Abstract

The novel concept of a microfluidic chip with an integrated three-dimensional fractal geometry with nanopores, acting as a gas transport membrane, is presented. The method of engineering the 3D fractal structure is based on a combination of anisotropic etching of silicon and corner lithography. The permeation of oxygen and carbon dioxide through the fractal membrane is measured and validated theoretically. The results show high permeation flux due to low resistance to mass transfer because of the hierarchical branched structure of the fractals, and the high number of the apertures. This approach offers an advantage of high surface to volume ratio and pores in the range of nanometers. The obtained results show that the gas permeation through the nanonozzles in the form of fractal geometry is remarkably enhanced in comparison to the commonly-used polydimethylsiloxane (PDMS) dense membrane. The developed chip is envisioned as an interesting alternative for gas-liquid contactors that require harsh conditions, such as microreactors or microdevices, for energy applications.

## 1. Introduction

The importance of membranes in microfluidic systems is reflected by numerous applications, including the detection of chemical reagents and gases, drug screening, cell culture, protein separation, chemical synthesis at the small-scale, and electrokinetic and hydrodynamic fluid transport [1,2]. In particular, when the role of the membrane is to act as a gas-liquid interface, effective gas absorption and minimal gas leakage is required [3]. The key issue for the combination of membranes and microfluidics is the sealing of the interfaces to avoid leakage, especially in the case of gases. The most convenient strategies to do this include (i) fabrication of the membrane as an integral part of the silicon chip; and (ii) exploiting the permeation properties of certain polymers by fabricating the chips directly from these materials [2]. In the case of biological applications this last option has been adopted in most of the cases by using polydimethylsiloxane (PDMS) chips; it is well known that this polymer possesses high oxygen permeability [4]. However, when chemical or temperature resistance is needed, the use of silicon chips is preferred.

In recent years, at the University of Twente, an elegant fabrication method has developed, which is based on a combination of anisotropic etching of silicon and so-called corner lithography, to create small nanoapertures of approximately 80–100 nm in the form of a three-dimensional fractal geometry [5,6]. Fractal geometry describes disciplines that consider symmetry-broken structures where, after a magnification, the shape appears identical. In other words, the magnified piece is almost a copy of the whole. The important features of the mentioned fabrication process are the possibility to easily scale up to the wafer-level and the ability to tailor the number of apertures and, thus, control the diffusion of gases through them. These apertures are distributed on the corners of pyramids which are part of a 3D fractal structure that can be replicated. This fact adds an additional advantage that could not be achieved in other 2D materials that are easily integrated with silicon microfluidics, such as porous silicon or anodized alumina. The 3D fractal structure can be embedded in a microchannel, which results in a larger interfacial area and a higher surface to volume ratio.

The hypothesis in the presented research is that the concept of membranes made of 3D fractals containing nanoapertures (also referred to as nanonozzles in this work) will result in effective gas permeation. To test this, the 3D fractal structure containing nanoapertures is integrated in a microfluidic channel and gas permeation through the pores is measured in order to quantify the diffusion of the gases oxygen and carbon dioxide through the fractal membrane. The measured values are validated with a model that considers the combination of the viscous and molecular flow regimes, the latter being the dominant mechanism for diffusion through the nanonozzles.

## 2. Materials and Methods

### 2.1. Integration of Fractals in Microchannels

**Figure 1:**
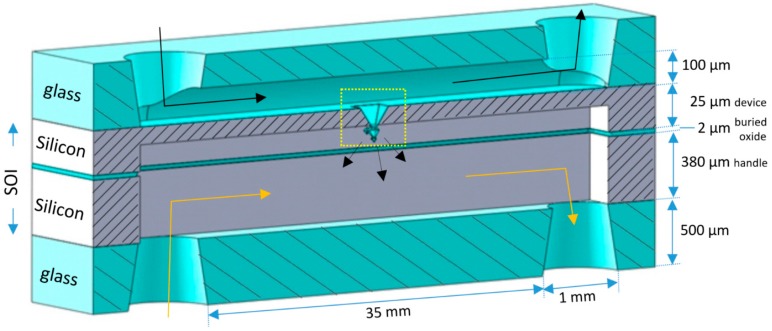
Conceptual representation of a chip with a fractal-based membrane for gas permeation.

In Figure 1 the schematic representation of a chip is shown in which fractals are used as the membrane between two microfluidic channels. The fractal membrane is made in a silicon-on-insulator (SOI) substrate, and microchannels are embedded in a glass substrate (the top wafer in Figure 1) and the SOI substrate. Below, the first general fabrication considerations are given, followed by more detailed process information.

Fractal structures are made in the device layer of the SOI substrate by means of anisotropic etching of silicon (Si) and corner lithography (CL) [6]. More specifically, for the chips, oxide-only corner lithography [7] is applied to create 3rd-generation fractals. Oxide-only CL utilizes thermal silicon oxide and selective isotropic etching of this layer. Due to the crystalline property of silicon, oxide-only CL, in combination with anisotropic silicon etching, results in the formation of octahedral cavities bounded by (111) Si-planes. A repetition of the sequence of oxide-only CL and Si-anisotropic etching yields the formation of 3D octahedral fractals of which the number of generations can be controlled. For the gas permeation chips the dimension of each subsequent generation of octahedral structures scaled down by a factor 2 has been chosen in order to obtain robust, well-defined, repetitive fractals. The realized chips contain a membrane with 3rd-generation fractals of which the apices are opened, yielding nanonozzles, in order to accomplish gas permeation. The gas channel above the fractal-based membrane is created in glass, whereas the gas channel below this membrane is realized in the handle-, buried oxide (BOX)-, and device layer of the SOI substrate. Inlets and outlets to both gas channels are fabricated in the top and bottom glass substrates.

**Figure 2:**
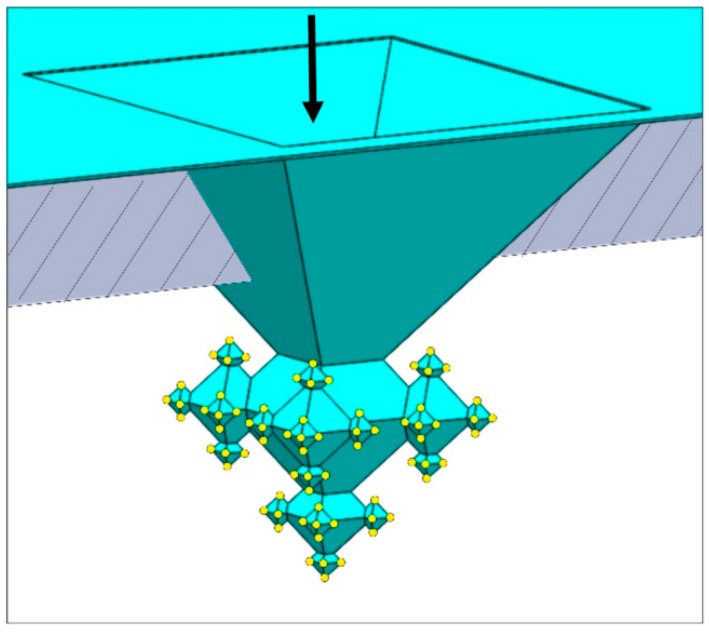
Schematic representation of the fabrication of a 3rd-generation fractal.

The starting points for the fractals (Figure 2) are inverted pyramids wet-etched in the 25 µm thick device layer of the SOI substrate. A 162-nm thick SiO_2_ mask (dry oxidation, 95 min at 1100 °C) patterned with UV lithography and BHF etching (6 min; giving openings with a diameter of 27 µm) served as mask for etching in potassium hydroxide (KOH; 25 wt %, 75 °C, etch time: 28.5 min). After KOH etching the SOI substrate was cleaned in RCA-2 (20 min; HCl:H_2_O_2_:H_2_O (1:1:5 vol %)), followed by the removal of the SiO_2_ mask (50% hydrofluoric acid (HF), 15 s). Subsequently, a three-fold execution of the sequence of oxide-only CL and anisotropic Si etching is performed to realize 3rd-generation fractals (Figure 2):

1. In the first step a ca. 163-nm thick SiO_2_ layer is deposited (dry oxidation, 95 min at 1100 °C), which is isotropically etched in 1% HF for 20.5 min upon which only in the corners of the inverted pyramids the underlying silicon are accessible (i.e., the (100) and (111) Si planes remain covered with SiO_2_ after this etch time). Then, 1st-generation octahedral structures are etched in silicon with tetramethylammonium hydroxide (TMAH; 25 wt %, 70 °C, 126 min). After TMAH etching the SiO_2_ layer is removed with 50% HF (18 s), followed by ozone/steam cleaning of the substrate.
2. In the second step, again, dry oxidation is used (95 min at 1100 °C), yielding a layer thickness of ca. 159 nm. Similar to the first step, this SiO_2_ film is isotropically thinned down in 1% HF (19.5 min), followed by anisotropic etching of silicon to create the 2nd-generation octahedral features (TMAH; 25 wt %, 70 °C, 65 min). Immersion of the SOI substrate in 50% HF (18 s) is carried out to remove the SiO_2_ mask, followed by ozone/steam cleaning to prepare the substrate for the next process step.
3. The third step also starts with dry oxidation (95 min at 1100 °C), giving a layer of ca. 160 nm, that is isotropically etched in 1% HF (20 min). The patterned film serves as selective mask during TMAH etching of silicon (25 wt %, 70 °C, 37 min) during which the 3rd-generation octahedrals are formed. Afterwards, the SiO_2_ film is stripped (50% HF, 15 s) and ozone/steam cleaning is performed.

**Figure 3:**
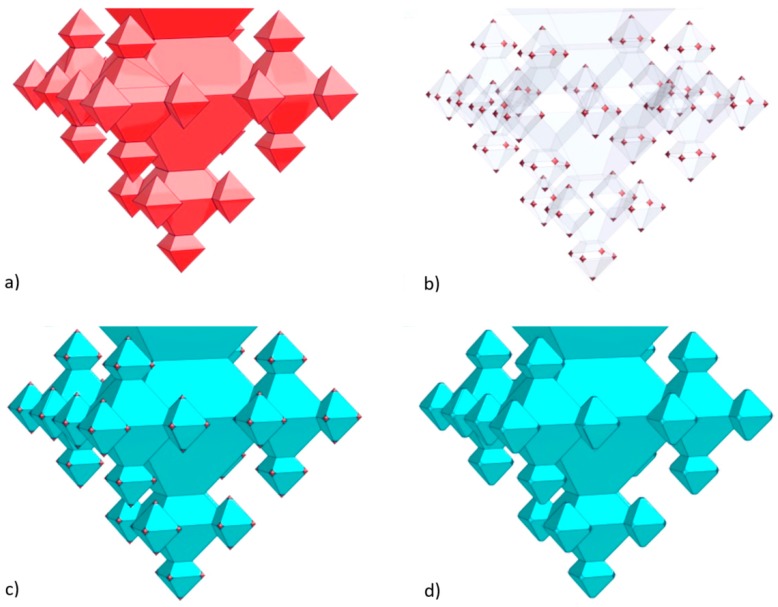
Schematic representation of the realization of apices/nanonozzles in 3rd-generation fractals (images reproduced with permission from [6]), resulting in a fractal-based gas permeation structure.

In order to accomplish gas permeation, the apices of the 3rd-generation fractals have to be opened, thus, nanonozzles have to be created. This is done by means of ‘conventional’ corner lithography, i.e., deposition and isotropic etching of silicon nitride and local oxidation of silicon (LOCOS) [5,6]. The concept is shown in Figure 3. On the 3rd-generation fractals a layer of 90 nm silicon nitride (Si_3_N_4_) is conformally deposited using low-pressure chemical vapour deposition (LPCVD) (Figure 3a)). This film is subsequently isotropically etched in hot phosphoric acid (85% H_3_PO_4_; 180 °C) for 22 min and 6 s (etch-factor 1.35), which leaves dots of Si_3_N_4_ in the corners of the fractal structure (Figure 3b)). After ozone/steam cleaning, LOCOS is performed (dry oxidation, 45 min at 1050 °C) yielding ca. 77 nm SiO_2_ on (111)-Si (Figure 3c)). The LOCOS is followed by an HF dip (1% HF, 30 s) to remove the (ultra)thin oxide from the Si_3_N_4_. Then the Si_3_N_4_ dots are selectively removed from the apices with H_3_PO_4_ (85% H_3_PO_4_; 180 °C), yielding the nanonozzles (the remaining SiO_2_ thickness measured on (111)-Si is 67 nm) (Figure 3d)). Each 3rd-generation fractal contains 125 nanonozzles which, at this point of the fabrication process, are still ‘hidden’ in the device layer.

**Figure 4:**
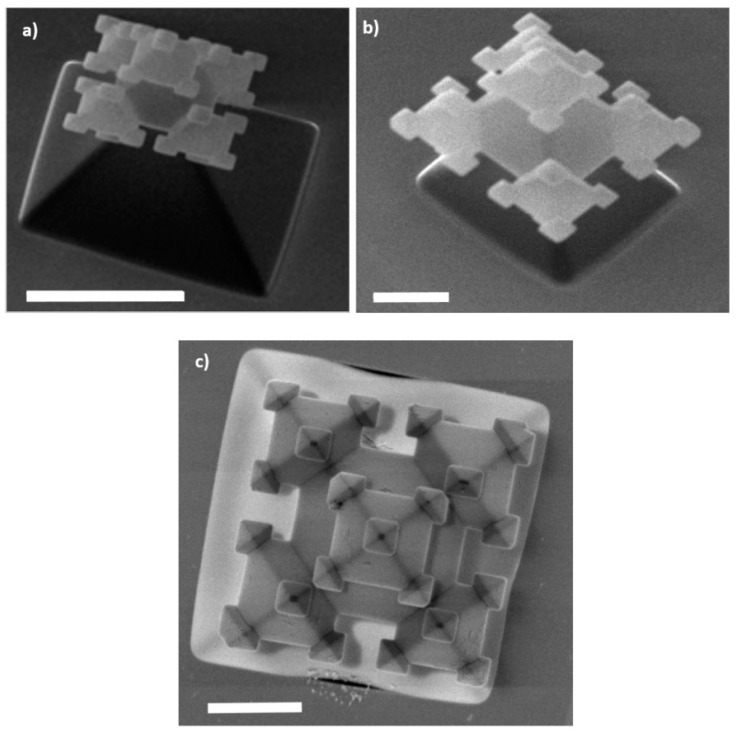
High-resolution SEM images of realized free-standing 3rd-generation fractals (embedded in the device layer of in the SOI substrate). It is noted that only in (**c**) are the nanonozzles are visible. Legend: in (**a**) the length of the scale bar is 5 µm, and in (**b**) and (**c**) the scale bar is 2 µm.

After the fabrication of fractals in the device layer of the SOI substrate, the device layer is processed. As can be seen in Figure 1, in this handle layer a gas channel is realized. This starts with a backside UV-lithography step (Olin 908-35 resist) in which the microfluidic channels are defined. This pattern is transferred in a 1-µm thick SiO_2_ layer (initial SiO_2_ layer thickness on the backside of handle layer is 2 µm, but 1 µm of SiO_2_ is consumed by the fractal fabrication process on the front side) by means of BHF etching (17 min). It is noted that during BHF etching of the backside of the SOI substrate the front side (containing the fractals) is protected with dicing foil (Nitto SWT10), which is removed after BHF etching. Deep reactive ion etching (DRIE), utilizing a pulsed SF_6_/C_4_F_8_ recipe at −40 °C, is applied to etch through the 380 µm thick handle layer. The buried oxide layer acts as an etch-stop for the DRIE. Subsequently, the 2-µm thick BOX layer is selectively removed with BHF (etch time 75 min; front side of SOI protected with SWT10 foil). Upon observing hydrophobicity in the etched gas channels, the dicing foil is peeled off and the photoresist is removed with an oxygen plasma, followed by Piranha cleaning (25 min; H_2_SO_4_:H_2_O_2_ (3:1 vol %)) of the SOI substrate. Then the device layer is slowly thinned from the bottom side by means of TMAH etching (25 wt %, 70 °C; etch rate ca. 280 nm/min), until the 3rd-generation fractals are released (and become free-standing in the gas channel). After 30 min the 3rd-generation SiO_2_ fractals are clearly visible (Figure 4).

As shown in Figure 1, the second gas channel is realized in a top glass substrate (500 µm thick MEMPax, Schott Glass, Penang, Malaysia). This is done using selective isotropic etching with 25% HF in combination with a lithographically-defined pattern in a multilayer of photoresist (Olin 907-17) and a sputtered Au/Cr film (120 nm/10 nm), which is described in detail elsewhere [8]. Fluidic accesses to this channel are realized with powder blasting [9]. This technique is also applied to create an inlet and outlet in a glass substrate that is used to seal the gas channel in the device layer of the SOI substrate (Figure 1).

Anodic bonding is carried out to bond the structured glass substrates to the processed SOI substrate [10]. First, the glass substrate containing the gas channel is bonded to the device layer of the SOI substrate (EVG501 bond system), followed by bonding of the bottom glass substrate to the assembled glass/SOI stack (using a home-built anodic bond system). The final step is the dicing of the bonded 100 mm diameter glass/SOI/glass stack into individual chips of 40 × 10 mm (DAD 321 Disco dicer; Disco HI-TEC Europe GmbH, Munich, Germany). During dicing both sides of the stack are covered with UV-curable dicing foil (Adwill D-210) to prevent dicing debris from entering the gas channels.

### 2.2. Chip Design and Assembly

The main considerations in the design of a proposed chip for gas diffusion in a channel are: (1) the number of fractal fabricated levels, which is directly connected with the number of openings; (2) the number of fractals in the channel; (3) the space between them; and (4) the distance from one phase to another, i.e., the depth of the channel, which is related to the diffusion distance.

**Figure 5:**
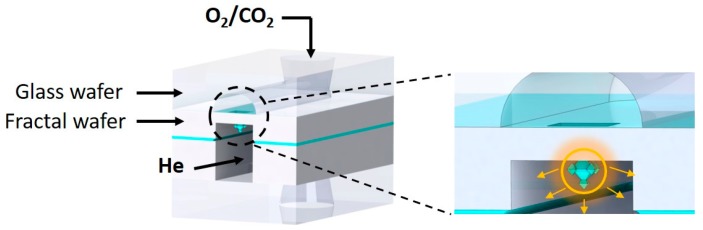
Schematic representation of the silicon fractal chip vertical cross-section.

The fractal structures were fabricated in a channel 3.5 cm long and 500 μm (Chip 1) or 300 μm (Chip 2) wide, containing a total of 244 (Chip 1) and 308 (Chip 2) 3rd-generation fractals unevenly distributed along the channel. Before the experiments, the total number of fractals containing opening pores was evaluated by optical microscopy; the final number of estimated pores and characteristics of the chips are presented in Table 1. The channel containing fractal structures was anodically bonded to a glass wafer with powder-blasted holes and assembled together with another wafer containing a single channel 380 μm deep and the same width and length as the fractal channels. Figure 5 shows a schematic representation of the final chip cross-section, containing a single free-hanging fractal for simplicity.

**Figure 6:**
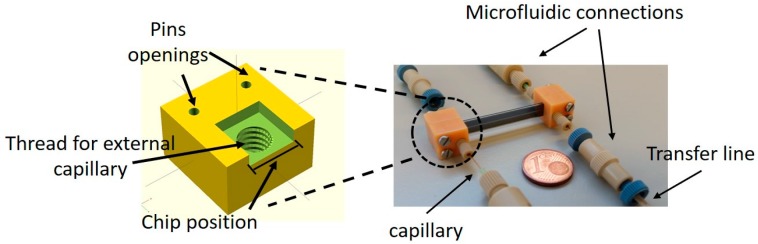
Device holder and fractal chip assembly.

To connect the microchip to external piping in order to be able to perform the permeation experiments, a fractal holder was designed and 3D printed (RapidShape GmbH, Heimsheim, Germany S30L). Four identical parts were printed from a photopolymer resin and the chip was sandwiched in between two parts at the inlet, and two parts at the outlet of the chip. The holder parts were connected by two pins of 1.5 mm diameter. Each fractal holder part consisted of a thread (3.95 mm diameter, 0.7938 threading and32 pitch) in order to connect the external capillary, 360 μm in diameter (Teknokroma, Tubing Fused Silica, Barcelona, Spain), through the Nanoport fittings (IDEX Health and Science, Nanoport Fittings, Oak Harbor, WA, USA) to enable the transfer line (3.2 mm in diameter) connection of the feed and sweep gases at the inlet, as well as the retentate and permeate gases at the outlet (see Figure 6).

### 2.3. Gas Permeation Measurements

**Figure 7:**
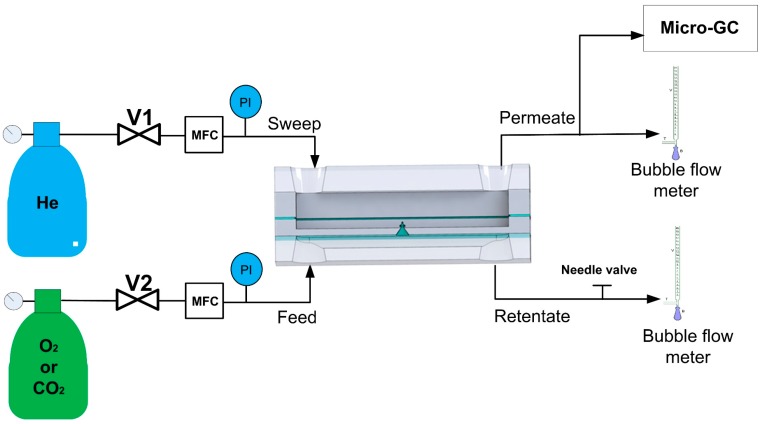
Experimental system for gas permeation.

The experimental system for gas permeation (see Figure 7) consists of: (1) gas sources; (2) mass flow controllers (Brooks, 5850 TR, Seattle, WA, USA); (3) pressure transducer (Panasonic, DP2-41E, Kadoma, Japan); (4) microfluidic fractal chip; and (5) micro-gas chromatograph (Micro-GC, Varian CP-4900, EVISA, Palo Alto, CA, USA). Two bubble meters were placed at the retentate and permeate sides, respectively, to ensure that the total flow inlet corresponds to the total flow outlet.

The permeation of two gases was measured: O_2_ (purity grade 99.999% Praxair, Zaragoza, Spain) and CO_2_ (high-purity grade 99.998% Praxair, Zaragoza, Spain). One of these two gases, pure, was introduced to the feed chamber at a given flow rate, controlled by the mass flow controller (MFC). The retentate was pressurized by a regulating valve located downstream (see Figure 7), and the pressure was measured by a pressure transducer (PI) at the entrance of the feed side. The flow of the pure gas was varied between 10 and 20 cm^3^ (STP)/min which resulted in a pressure in the feed side of 21.7 to 2.7 × 10^5^ Pa. The pressure drop along the retentate channel was calculated by solving the Navier-Stokes equation in COMSOL Multiphysics^®^ software and ranges from 3.5 to 6.8 × 10^3^ Pa. The sweep gas (He, purity grade 99.999% Praxair, Danbury, CT, USA) was introduced to the permeate chamber of the fractal microfluidic chip at a constant flow rate, 10 cm^3^ (STP)/min, and the pressure measured at the entrance was 1.1 × 10^5^ Pa. The pressure drop along the permeate channel was also calculated and corresponds to 95 Pa and 263 Pa for Chip 1 and Chip 2, respectively. The outlet of the permeate side was maintained at atmospheric pressure. Thus, the Δ*P* (driving force) between two chambers was in the range of 0.6 to 1.6 × 10^5^ Pa. The gas permeated through the nanoapertures to the permeate side and the mixture He + gas (CO_2_ or O_2_) was analysed in the micro-GC (Varian CP-4900) equipped with two modules, one with a M5A mole-sieve column and the other with Pora PLOT Q (PPQ) column. He was used as a carrier gas in both columns. The micro-GC was calibrated in the range of 2.5% to 4.5% in volume for CO_2_ and O_2_.

The mass transport coefficient, permeance, was calculated as the molar flow of the permeating gas per unit of membrane area divided by the driving force, according to Equation (1):

(1) $$P_{i}=\frac{Q_{perm}Y_{i}}{A\Delta P_{i}}$$

where *P_i_* is the permeance of the gas (i.e., O_2_ or CO_2_) [mol/m^2^·s·Pa], *Q_perm_* is the total molar flow of the permeate [mol/s], *Y_i_* is the molar fraction in the permeate side, *A* is the area of the membrane (see Table 1), and Δ*P_i_* corresponds to the driving force for permeation of “*i*” species and is the difference between the partial pressure in the feed side and the permeate side, respectively.

## 3. Results and Discussion

**Figure 8:**
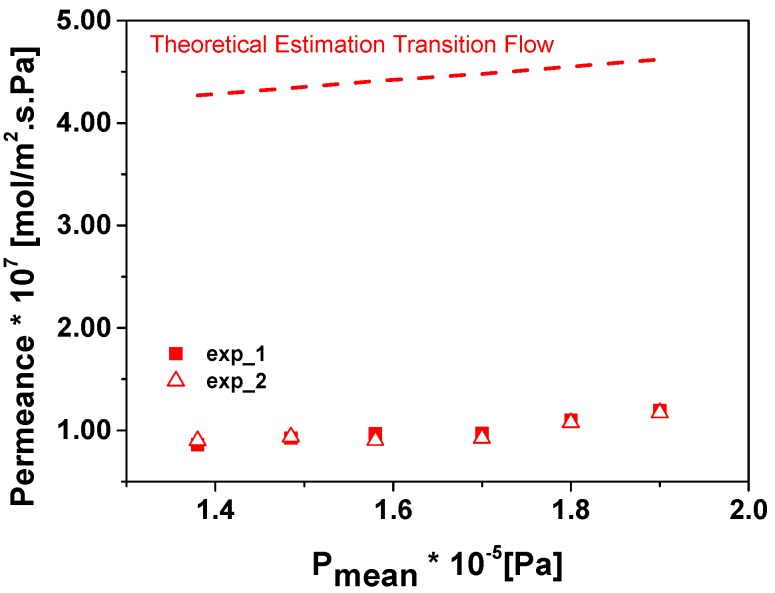
Experimental and theoretical permeation of O_2_ in Chip 1.

**Figure 9:**
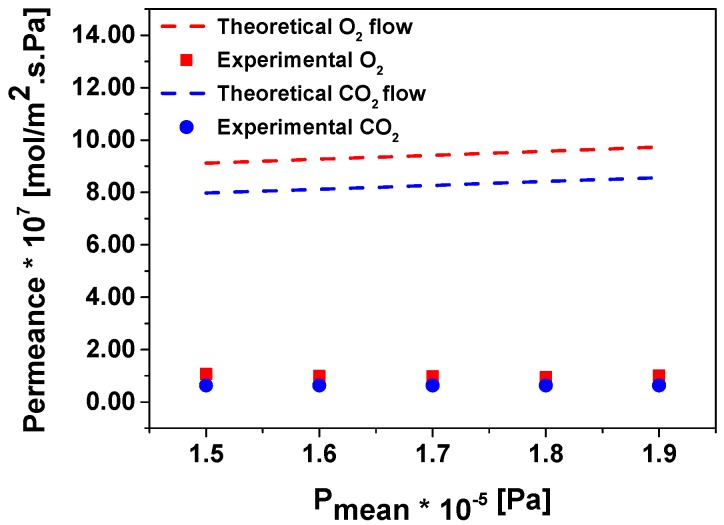
Experimental and theoretical permeation of O_2_ and CO_2_ in Chip 2.

Figure 8 shows two different sets of experimental points obtained for Chip 1 for permeation of oxygen versus mean pressure, calculated as the average between the total pressure in the feed side and permeate side, and Figure 9 corresponds to the permeation of oxygen and carbon dioxide in Chip 2. To evaluate the permeation through the pores of the fractal geometry two possible permeation mechanisms occurring simultaneously can be considered: molecular flow (or Knudsen) and viscous flow (or Poiseuille) [11,12]. All the permeation experiments show that there is no significant increase in the permeance with the mean pressure. Thus, as a first approximation, the viscous flow through these small nanoapertures could be considered negligible versus Knudsen flow [13].

The different flow regimes can be described by the dimensionless Knudsen number: Kn = *λ*/*d*, *λ* being the mean free path of the molecule and d the diameter of the pore. According to the Kn number, the gas flow behaviour can be divided into viscous (Kn < 0.01), transition (0.01 < Kn <1) and molecular (Kn > 1) flow regime. In our case, the Knudsen number is between 0.76 and 0.84, which indicates that we are dealing with transition flow.

The transition flow through an ultrathin nanosieve membrane on top of a microsieve membrane, (see Figure 5) was described by Unnikrishnan et al. as a linear addition of viscous and molecular fluxes [12]. The nanosieve membrane had a thickness of 45 nm with circular nanopores of 120 nm supported on top of a microsieve membrane made of straight cylindrical pores of 6 μm, with a length of 80 μm. The flow through the membrane was described as a series resistance model where the total pressure drop (Δ*P_total_* [Pa]), i.e., the resistance, is the sum of the pressure drop through the micropores (Δ*P_micro_* [Pa]), and the pressure drop through the nanopores (Δ*P_nano_* [Pa]), see Equation (2)). The total flow (*Φ_total_* [mol/s]), is the same as the flow through the micropores (*Φ_micro_* [mol/s]), and the flow through the nanopores (*Φ_nano_* [mol/s]), due to the conservation of mass (see Equation (3)):

(2) $$\Delta P_{total}=\Delta P_{micro}+\Delta P_{nano}$$

(3) $$\Phi_{total}=\Phi_{micro}=\Phi_{nano}$$

Then, the transition flux is a linear addition of the viscous and molecular fluxes:

(4) $$\Phi_{nano}=\Delta P_{nano}\left(F_{i-viscous}+F_{i-molecular}\right)$$

where *F_i_* represents the flow conductance, [mol/s Pa] viscous or molecular, either through the micropores or nanopores, that is represented by the following equations; in the case of molecular flow through the nanopores:

(5) $$F_{i-molecular}=\frac{A\varepsilon }{\sqrt{2\pi MRT}}{\left(1+\frac{t}{d}\right)}^{-1}$$

where *F_i-molecular_* is the flow conductance in the molecular flow regime through nanopores, i.e., [mol·s^−1^·Pa^−1^], *A* is the total membrane surface area, see Table 1 [m^2^], *ε* is the porosity, *M* is the gas molecular weight [kg·mol^−1^], *R* is the gas constant [J·mol^−1^·K^−1^], and *T* is the temperature [K]. The term (1 + *t*/*d*), where *t* is the thickness of the pore and *d* the diameter of the pore, is the Clausing function that considers the collisions of gas molecules with the walls of the pore and it is related to its geometry [14].

In the case of viscous regime, the flow conductance can be described by:

(6) $$F_{viscous}=\frac{A\varepsilon rP}{3\eta RT\pi }{\left(1+\frac{8t}{3\pi r}\right)}^{-1}\left(1-f\left(\varepsilon \right)\right)$$

where *P* is the arithmetic mean pressure, and *η* is the viscosity [Pa s] of the gas used, *t* is the thickness of the pore and *r* is the radius of the pore. The term (1 + 8*t*/3*πr*) describes the frictional losses experienced by the gas due to interaction with the pore surface and (1 − *f*(*ε*)) quantifies the influence of flow through the neighbouring pores on the flow through a single pore and, in the case of the straight cylindrical pores, was estimated as 0.9743 for the nanosieves [12].

The system that we have to simulate is more complex compared to the one presented by Unnikrshnan et al. Our system has a 3D structure made based on 3rd-generation fractals emerging from the 2D membrane (SOI-wafer). Additionally, it is difficult to estimate the real size of all nanoapertures due to the fact that some could be closed, or only partially opened. Thus, to apply the model described above we have made several assumptions and simplifications, to obtain an estimation of the theoretical flux useful for a preliminary validation of our experimental data. We represent our membrane system in a similar way, as in the case of the ultrathin nanosieve [12], made of micropores and nanopores. According to IUPAC, the apertures in the porous membranes can be divided into three main groups regarding their size: micropore (pore size not exceeding 2 nm), mesopore (size in the range of 50 nm to 0.05 μm), and macropore (larger than 0.05 μm). However, following the nomenclature used in the article of Unnikrshnan et al. [12], in our case the micropores correspond to the different fractal generations of micrometer size and the nanopores correspond to the 3rd-generation nanonozzles with a thickness *t* = 67 nm (the remaining SiO_2_ after removing Si_3_N_4_, see Section 2.1) and an average diameter *d* = 100 nm. Considering the size of the micropores, in the range of 1 to 20 microns, we assume that the pressure drop accounts only through the nanoapertures. The porosity of the chip was calculated as the ratio of the total open nanopore area over the total channel area (see Table 1).

The calculated values for the transition flow in the case of Chip 1 are presented as dashed lines in Figure 8. The estimation of the permeance is around 4 times higher in the case of Chip 1 and 9 times for Chip 2 (not presented in Figure 9). These differences could be attributed to the difficulties in the estimation of the real size of the pore apertures, which could vary from 50 to 150 nm. The theoretical values show that the contribution of the viscous flow to the total flow is between 14% and 20% depending on the mean pressure. This agrees well with our initial observation about the importance of the molecular flow over viscous. In case of the Knudsen diffusion mechanism, the permeation of the gases depends on the molecular weight and is inversely proportional to the square root of *M* (see Equation (5)). Thus, diffusion of smaller molecules is faster compared to larger molecules and, accordingly, the ideal selectivity of O_2_ over CO_2_ could be calculated as the square root of 44 over 32, which results in a value of 1.2, whereas we found a slightly higher value of 1.6 in our experimental data (Figure 9).

The permeation values obtained, in the order of 10^−7^ mol/m^2^·s·Pa, are high in comparison with polymeric materials, such as polydimethylsiloxane (PDMS). Considering a permeability value for PDMS of 620 Barrer for oxygen [4], a membrane film of just 1–4 micrometers would be required to obtain the same permeation flux as the nanonozzles presented here. However, such thin PDMS membranes cannot be handled. Table 2 shows the experimentally-obtained fluxes of O_2_ and CO_2_ in two chips as a function of P_mean_ and their corresponding theoretical thicknesses of the PDMS film.

The gas permeation of a fractal membrane is worse than, for example, anodized alumina membranes with 200 nm pores {Cooper, 2003 #30}. However, it would be very difficult and time consuming to integrate alumina membranes in the microfluidic chip. Moreover, there is a risk of membrane breaks or cracks in the presence of high pressure and leaks through the system. Depending on the reagents, the fabrication of anodized alumina can be very expensive.

## 4. Conclusions

In this work, we have validated the concept of a new 3D membrane structure incorporated in a chip for gas transfer between two microfluidic channels. We experimentally measured and theoretically calculated, considering a simplified model and several assumptions, the permeation of oxygen and carbon dioxide. The chip is characterized by portability, increased contact surface, and mechanical stability since the membrane is embedded in a silicon wafer. Therefore, there is no risk of thin film deflection or material swelling. Moreover, due to the compactness of the system, the distance for diffusion was extremely reduced which resulted in the decreased travelling time and path of the molecules. This approach could work as an alternative to PDMS membranes since the permeation flux obtained through the nanonozzles in the 3D fractals is high.

Other applications apart from the single gas permeation tested here include gas-liquid contactors, where the 3D structure presents an advantage due to the higher surface-to-volume ratio compared to 2D membranes, and also applications requiring high temperatures and gas reactant distribution or product removal, such as micromembrane microreactors [15].

## Figures and Tables

**Table 1:** Properties of the fabricated fractal chips.

	Chip 1	Chip 2
Channel width (μm)	500	300
Number open fractal structures	244	308
Si membrane area, *A* (m^2^)	1.75 × 10^−5^	1.05 × 10^−5^
Porosity, *ε*	1.37 × 10^−5^	2.88 × 10^−5^

**Table 2:** O_2_ and CO_2_ permeation fluxes through the 3D fractal nanonozzles as a function of mean pressure and corresponding theoretical PDMS membrane thickness.

P_mean_·10^−5 ^[Pa]	P_O2_ [mol/m^2^·s·Pa]		Equivalent PDMS Thickness [μm]	P_CO2_ [mol/m^2^·s·Pa]	Equivalent PDMS Thickness [μm]
	Chip 1	Chip 2	O_2_	Chip 2	CO_2_
1.38	8.79 × 10^−8^	1.02 × 10^−7^	1.12	6.48 × 10^−8^	4.1
1.48	9.31 × 10^−8^	1.06 × 10^−7^	1.07	6.31 × 10^−8^	4.2
1.58	9.34 × 10^−8^	9.88 × 10^−8^	1.09	6.10 × 10^−8^	4.3
1.7	9.48 × 10^−8^	9.85 × 10^−8^	1.08	6.46 × 10^−8^	4.1
1.8	1.08 × 10^−7^	9.46 × 10^−8^	1.00	6.26 × 10^−8^	4.2
1.9	1.18 × 10^−7^	1.01 × 10^−7^	0.93	5.96 × 10^−8^	4.4
